# Spectacular and Prompt Response to Extracorporeal Photopheresis for Refractory Cutaneous Chronic Graft-Versus-Host Disease after Allogeneic Hematopoietic Stem Cell Transplantation: A Case Report

**DOI:** 10.3390/medicina58121722

**Published:** 2022-11-24

**Authors:** Adrianna Spałek, Iwona Grygoruk-Wiśniowska, Karolina Gruenpeter, Marta Panz-Klapuch, Grzegorz Helbig

**Affiliations:** Department of Hematology and Bone Marrow Transplantation, Faculty of Medicine in Katowice, Medical University of Silesia, 40-055 Katowice, Poland

**Keywords:** allogeneic hematopoietic stem cell transplantation, extracorporeal photopheresis, graft versus host disease, immunosuppressive drugs

## Abstract

Chronic graft-versus-host disease (cGVHD) is a serious complication after allogenic hematopoietic stem cell transplantation (allo-HSCT), negatively affecting the morbidity and mortality of recipients. Skin involvement is the most common cGVHD manifestation with a wide range of pleomorphic features, from scleroderma to ulcerations and microangiopathic changes. Despite the access to many immunosuppressive drugs, therapy for cGVHD is challenging. Systemic steroids are recommended as the first-line treatment; but, in steroid-resistant patients, extracorporeal photopheresis (ECP) remains one of the subsequent therapeutic options. Here, we present a case report of a 31-year patient suffering from advanced steroid-refractory skin and oral mucosa cGVHD who was spectacularly treated with ECP. It was the first time we observed such “overnight” resolution of the graft-versus-host disease syndrome. The present report proves the important role of ECP in the treatment of steroid-resistant cGVHD, especially when other immunosuppressive therapies have failed.

## 1. Introduction

Graft-versus-host disease (GVHD) is one of the leading causes of morbidity and mortality after allogeneic hematopoietic stem cell transplantation (allo-HSCT) [1]. Its pathogenesis is associated with inflammation, immune-mediated organ injury, and subsequent fibrosis [2]. Chronic GVHD (cGVHD) usually develops months after allo-HSCT, but it sometimes may occur as a prolongation of prior acute manifestation. Chronic GVHD may affect every organ, but skin involvement remains the most frequent manifestation with a wide range of pleomorphic features, from scleroderma to lichen planus-like eruption, depigmentation, microangiopathic changes, and poikiloderma [3].

Despite the wide access to many immunosuppressive drugs, the treatment of cGVHD remains a challenge. Steroids are still the first-line treatment; but, in steroid refractory patients, extracorporeal photopheresis (ECP) remains one of the subsequent therapeutic choices [4]. It is a leukapheresis-based immunomodulatory therapy in which collected lymphocytes are exposed to ultraviolet-A radiation in the presence of the photosensitizing agent, 8-methylpsoralen, and then reinfused to the patient [5]. The efficacy of ECP in cGVHD is well-documented with an overall response rate varying from 31% to 93%, and the highest efficacy is noted for those with skin involvement [6]. Of note is that prolonged ECP is usually needed for evident visual effect [2,6].

Here, we present the case of a 31-year-old patient suffering from steroid-refractory skin and oral mucosa cGVHD who promptly and spectacularly responded to ECP.

## 2. Case Report

A 31-year-old male patient underwent allo-HSCT from a 9/10 HLA-matched male unrelated donor for acute myeloid leukemia (AML) in June 2021. He was diagnosed with AML and translocation t(9;11) six months earlier. The patient received induction chemotherapy consisted of daunorubicin and cytarabine. As a consequence, he achieved complete remission (CR) with negative measurable residual disease (MRD). The remission was consolidated by two cycles of high-doses of cytarabine. As a myeloablative conditioning, busulfan and cyclophosphamide were provided. For GVHD prophylaxis, he received cyclosporine with methotrexate as well as anti-thymocyte globulin (Thymoglobulin).The transplantation procedure as well as the early post-transplant period were unremarkable, and the patient was found to have full donor chimerism at discharge. CR was confirmed using repeated bone marrow examination, and cyclosporin was gradually reduced till complete withdrawal five months after allo-HSCT.

**Figure 1:**
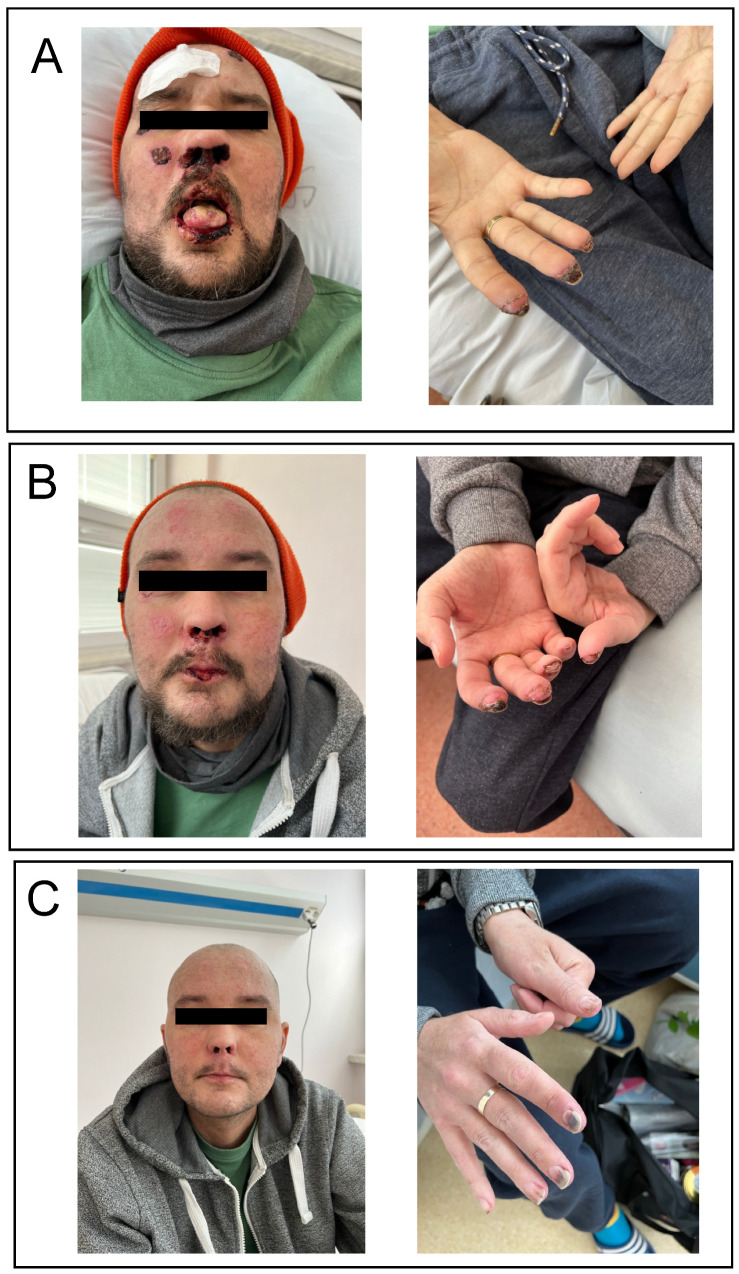
Favourable effects of therapy with extracorporeal photopheresis (ECP) to treat skin and mucosa advanced chronic graft-versus-host disease (cGVHD): (**A**) presentation of symptoms before treatment (+304 day post allo-HSCT); (**B**) partial regression after two ECP procedures (+310 day post allo-HSCT); and (**C**) almost complete response after four ECP procedures (+326 day post allo-HSCT).

On day +212 after transplantation, while being off immunosuppressants, the patient was urgently admitted for progressive weakness, watery diarrhea (up to 3000 mL of stool daily), severe abdominal pain, and nausea. Blood count was within the normal limits. In biochemistry, hyperbilirubinemia (60 µmol/L; normal range 3.4–20.5) with elevated levels of transaminases and alkaline phosphatase was detected. Clostridium difficile as well as other bacterial, viral, and fungal infections were excluded. Finally, he was diagnosed with late onset acute GVHD grade 4 according to the MAGIC classification [7]. The patient received methylprednisolone (MP) at 2 mg/kg daily in combination with mycophenolate mofetil (MMF) 500 mg twice a day, but the symptoms deteriorated. The doses of MP were gradually diminished, MMF was discontinued, and ruxolitinib (RUX) was added at 5 mg twice a day. Due to the lack of response, Thymoglobulin at a total dose of 6.5 mg/kg was given on day +230 after allo-HSCT. As a result, normalization of serum bilirubin concentration and complete regression of gastrointestinal symptoms were observed. At the same time, symptoms of cGVHD affecting the skin as well as nasal and oral mucosa gradually developed. The patient was discharged on RUX, low doses of MP, and oral methotrexate once weekly. Despite the triple immunosuppressive therapy, cutaneous and oral manifestations progressed. Ten months after transplantation, the patient developed ulcerations with the presence of multiple necrotic foci as well as microangiopathic changes on face, oral mucosa, and distal phalanges (Figure 1A). Painful lesions in the mouth made it impossible to eat. Differential diagnosis excluded transplant-associated thrombotic microangiopathy and other possible causes of the present changes. He was diagnosed with severe cGvHD [8]. Due to pancytopenia, methotrexate was stopped and the patient started ECP while on low doses of MP and RUX. First, two procedures were performed on day +308 and +309 after allo-HSCT. Surprisingly, the visual effect of treatment was observed immediately on the next day (as presented on Figure 1B). The patient also reported an improvement in general condition and a reduction of pain. The continuation of ECP resulted in further resolution of skin and mucosa lesions (Figure 1C). Due to the reported favourable effects of RUX + ECP combination [9], the treatment was continued for six months. In total, the patient received eight ECP procedures which led to a complete deterioration of cGVHD symptoms. The dosage of RUX was diminished to 5 mg per day due to the occurrence of grade 3 thrombocytopenia. There were no significant infectious complications after the initiation of ECP treatment. At the time of observation, the patient remained in complete hematologic remission with negative MRD and full donor chimerism.

## 3. Discussion

The efficacy of ECP in cGVHD is well-established and reaches up to 70% in skin manifestation and 63% in oral mucosa, but the efficacy differs between studies [10]. Effective immunosuppressive treatment with ECP enables a decrease in steroid dose of more than 50%, which affects overall survival [11]. The procedures should be performed on two consecutive days every week and continued for at least eight cycles until there is a noticeable response [12]. The time till initial response to ECP differs between reports, but none of them describes an immediate improvement as presented in our case. The median time to response was 26 days in a paper by *Couriel* et al. [13]; however, it might be shorter [11].

Of note is that the differential diagnosis of microangiopathic changes presented in our patient includes TA-TMA, although GVHD may also play a role in the pathogenesis of this complication [14]. There is significant diversity among the diagnostic criteria of TA-TMA, but those presented by *Jodele* et al. [15] incorporate the recent findings in the field: (1) presence of schistocytes on blood smear; (2) elevated serum LDH; (3) proteinuria ≥ 30 mg/dL or hypertension; (4) de novo thrombocytopenia; (5) de novo anemia; and (6) elevation of terminal complement- sC5b-9 [14]. Although the measurement of terminal complement activation was not available in our center, our patient did not present any other abovementioned diagnostic criteria. It had been suggested that levels of sC5b-9, extracellular deoxyribonucleic acid-myeloperoxidase (DNA-MPO), and thrombin–antithrombin complex should be determined [16]. Higher levels of those parameters were detected in patients with TA-TMA when compared to GVHD patients [17].

One should remember that some drugs may have an impact on the development of microangiopathic changes. Calcineurin inhibitors, including cyclosporin A, tacrolimus, and sirolimus have been proven to increase the risk of TA-TMA, while steroids and MMF seem to be relatively safe in this aspect [18].

The exact mechanism of ECP in cGVHD is not well-understood. Some data suggest that ECP-induced T-cell tolerance depends on T-cell apoptosis and induction of regulatory T-cells [19]. The role of B-cells is also taken under consideration, but it requires further studies [20].

## 4. Conclusions

To the best of our knowledge, an “overnight” improvement of cGVHD after ECP has never been reported before. ECP may remain a valuable therapeutic option, especially for patients with skin and oral manifestation. The treatment with ECP should be initiated as soon as possible in those with steroid refractoriness.

## Data Availability

The data used in the current study are available from the corresponding author upon reasonable request.
